# *N*,*N*-Dimethyl-*N*′,*N*′′-bis­(2-methyl­phenyl)phospho­ric triamide mono­hydrate

**DOI:** 10.1107/S1600536812033995

**Published:** 2012-08-04

**Authors:** Farnaz Eslami, Mehrdad Pourayoubi, Mohammad Yousefi, Arnold L. Rheingold, James A. Golen

**Affiliations:** a Department of Chemistry, Shahr-e Rey Branch, Islamic Azad University, Tehran, Iran; b Department of Chemistry, Ferdowsi University of Mashhad, Mashhad, Iran; c Department of Chemistry, University of California, San Diego, 9500 Gilman Drive, La Jolla, CA 92093, USA

## Abstract

In the title compound, C_16_H_22_N_3_OP·H_2_O, the P atom adopts a distorted tetra­hedral environment with the bond angles around the P atom in the range 99.98 (7)–116.20 (7)°. The P—N bond length in the [(CH_3_)_2_N]P(O) fragment [1.6392 (14) Å] is slightly shorter than two other P—N bonds [1.6439 (15) and 1.6530 (14) Å]. In the (CH_3_)_2_NP(O) fragment, one of the methyl groups is *syn* to the P=O bond, whereas the other one is *anti* to the P=O bond [C—N—P=O torsion angles = 4.80 (17) and −174.57 (15)°]. In the crystal, the water mol­ecules form hydrogen bonds to the O atoms of the P=O bond of two different mol­ecules and act as acceptors for the two amino H atoms of the same mol­ecule. As a result, chains parallel to [010] are formed.

## Related literature

For phospho­ramidates having a [(CH_3_)_2_N]P(O) fragment and for P=O and P—N bond lengths, see: Pourayoubi, Tarahhomi *et al.* (2012 ▶); Pourayoubi *et al.* (2011 ▶). For the double H-atom acceptor capability of the P=O group, see: Pourayoubi, Nečas & Negari (2012 ▶).
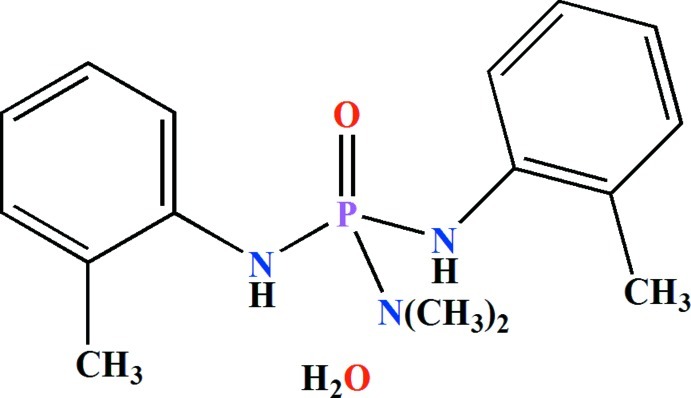


## Experimental

### 

#### Crystal data

C_16_H_22_N_3_OP·H_2_O
*M* *_r_* = 321.35
Monoclinic, 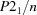

*a* = 10.7058 (16) Å
*b* = 7.2541 (11) Å
*c* = 22.091 (3) Å
β = 90.971 (2)°
*V* = 1715.3 (4) Å^3^
*Z* = 4
Mo *K*α radiation
μ = 0.17 mm^−1^
*T* = 100 K
0.20 × 0.14 × 0.14 mm

#### Data collection

Bruker APEXII CCD diffractometer
Absorption correction: multi-scan (*SADABS*; Bruker, 2008 ▶) *T*_min_ = 0.967, *T*_max_ = 0.977
15201 measured reflections
4036 independent reflections
3135 reflections with *I* > 2σ(*I*)
*R*_int_ = 0.050

#### Refinement

*R*[*F* ^2^ > 2σ(*F* ^2^)] = 0.043
*wR*(*F* ^2^) = 0.115
*S* = 1.04
4036 reflections
215 parameters
5 restraints
H atoms treated by a mixture of independent and constrained refinement
Δρ_max_ = 0.30 e Å^−3^
Δρ_min_ = −0.38 e Å^−3^

### 

Data collection: *APEX2* (Bruker, 2007 ▶); cell refinement: *SAINT* (Bruker, 2007 ▶); data reduction: *SAINT*; program(s) used to solve structure: *SHELXS97* (Sheldrick, 2008 ▶); program(s) used to refine structure: *SHELXL97* (Sheldrick, 2008 ▶); molecular graphics: *Mercury* (Macrae *et al.*, 2008 ▶); software used to prepare material for publication: *SHELXL97* and *enCIFer* (Allen *et al.*, 2004 ▶).

## Supplementary Material

Crystal structure: contains datablock(s) I, global. DOI: 10.1107/S1600536812033995/bt5974sup1.cif

Structure factors: contains datablock(s) I. DOI: 10.1107/S1600536812033995/bt5974Isup2.hkl

Additional supplementary materials:  crystallographic information; 3D view; checkCIF report

## Figures and Tables

**Table 1:** Hydrogen-bond geometry (Å, °)

*D*—H⋯*A*	*D*—H	H⋯*A*	*D*⋯*A*	*D*—H⋯*A*
O1*W*—H2*W*⋯O1^i^	0.84 (1)	1.91 (2)	2.7491 (17)	173 (2)
O1*W*—H1*W*⋯O1^ii^	0.85 (1)	1.91 (1)	2.7607 (17)	175 (2)
N1—H1*N*⋯O1*W*	0.87 (1)	2.04 (2)	2.8724 (19)	159 (2)
N2—H2*N*⋯O1*W*	0.87 (1)	2.00 (2)	2.8473 (18)	164 (2)

Symmetry codes: (i) 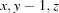; (ii) 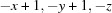.

**Fig. 1.**
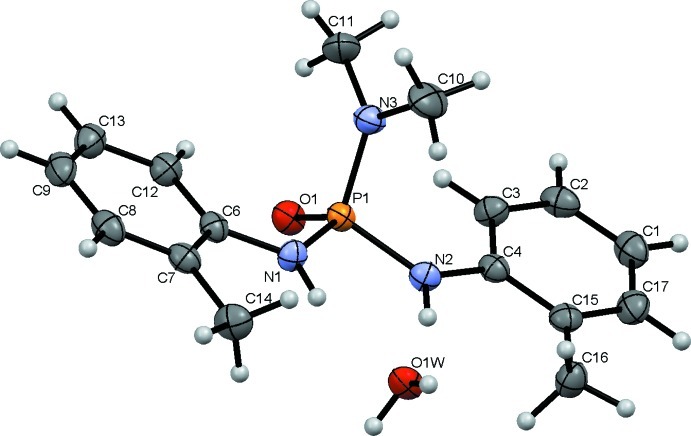
An ORTEP-style plot and atom labeling scheme for the title hydrate compound. Displacement ellipsoids are given at 50% probability level and H atoms are drawn as small spheres of arbitrary radii.

**Fig. 2.**
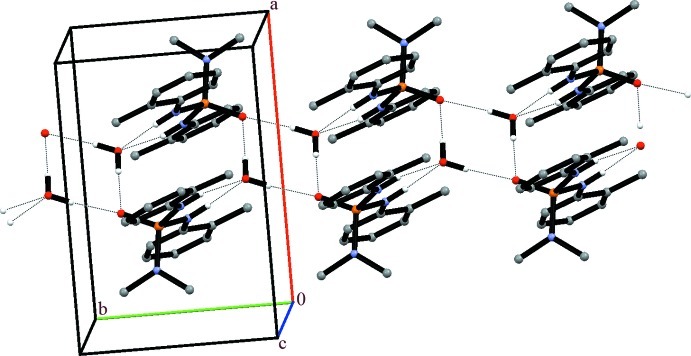
Packing in the title compound with hydrogen bonds shown as dotted lines. Only H atoms involved in hydrogen bonds are shown.

## supplementary crystallographic information

### Comment

The crystal structure determination of the title hydrate phosphoric triamide (Fig. 1) was performed as a part of work on synthesis and X-ray crystallography of compounds with a [(CH_3_)_2_N]P(O) fragment (Pourayoubi, Tarahhomi *et al.*, 2012; Pourayoubi *et al.*, 2011).

In the phosphoric triamide molecule, the P atom adopts a distorted (N)P(O)(N)_2_ tetrahedral environment. The P═O and P—N bond lengths are within the expected values (Pourayoubi, Tarahhomi *et al.*, 2012; Pourayoubi *et al.*, 2011). The sum of three bond angles at the nitrogen atom of the dimethylamido fragment, C11—N3—C10 + C11—N3—P1 + C10—N3—P1, of 360° suggests *sp*^2^ hybridization for this N atom. Moreover, the C6—N1—P1 and C4—N2—P1 bond angles are 125.07 (12)° and 125.66 (12)°, respectively. The P—N bond length of the [(CH_3_)_2_N]P(O) fragment is shorter than two other P—N bonds.

In the crystal, the oxygen atoms of phosphoryl group and water molecule act as double-hydrogen bond acceptors (for a definition of double-hydrogen bond acceptor, see: Pourayoubi, Nečas & Negari, 2012) to form O—H···O···H—O and N—H···O···H—N groups. The phosphoric triamide and water molecules are aggregated through these hydrogen bonds in a linear arrangement parallel to the *b* axis, Fig. 2.

### Experimental

Synthesis of ((CH_3_)_2_N)P(O)Cl_2_: [(CH_3_)_2_NH_2_]Cl (0.184 mol) and P(O)Cl_3_ (0.552 mol) were refluxed for 8 h and afterwards the excess of P(O)Cl_3_ was removed *in vacuo*.

Synthesis of title compound: To a solution of ((CH_3_)_2_N)P(O)Cl_2_ (3.7 mmol) in CHCl_3_ (15 ml), a solution of *ortho*-toluidine (14.8 mmol) in the same solvent (25 ml) was added at 273 K. After 4 h stirring, the solvent was removed and product was washed with deionized water and recrystallized from chloroform/*n*-hexene at room temperature to yield colourless crystals.

### Refinement

The H1N, H2N, H1W and H2W atoms were found from a Fourier difference map and their coordinates were refined with the following restraints: N—H = 0.87 (2) Å, O—H = 0.85 (2) Å and H1W···H2W = 1.33 (2) Å. Their displacement parameters were set to 1.2 *U*_eq_ of the parent atom. All other hydrogen atoms were placed in calculated positions and allowed to ride on their parent C atoms; C—H distances (CH_3_) 0.98 Å, (CH) 0.95 Å with *U*_eq_ of 1.5 and 1.2, respectively.

### Crystal data

**Table:**

C_16_H_22_N_3_OP·H_2_O	*F*(000) = 688
*M**_r_* = 321.35	*D*_x_ = 1.244 Mg m^−^^3^
Monoclinic, *P*2_1_/*n*	Mo *K*α radiation, λ = 0.71073 Å
Hall symbol: -P 2yn	Cell parameters from 4384 reflections
*a* = 10.7058 (16) Å	θ = 3.0–28.1°
*b* = 7.2541 (11) Å	µ = 0.17 mm^−^^1^
*c* = 22.091 (3) Å	*T* = 100 K
β = 90.971 (2)°	Block, colourless
*V* = 1715.3 (4) Å^3^	0.20 × 0.14 × 0.14 mm
*Z* = 4	

### Data collection

**Table:**

Bruker APEXII CCD diffractometer	4036 independent reflections
Radiation source: fine-focus sealed tube	3135 reflections with *I* > 2σ(*I*)
Graphite monochromator	*R*_int_ = 0.050
φ and ω scans	θ_max_ = 28.4°, θ_min_ = 1.8°
Absorption correction: multi-scan (*SADABS*; Bruker, 2008)	*h* = −14→13
*T*_min_ = 0.967, *T*_max_ = 0.977	*k* = −9→9
15201 measured reflections	*l* = −29→29

### Refinement

**Table:**

Refinement on *F*^2^	Primary atom site location: structure-invariant direct methods
Least-squares matrix: full	Secondary atom site location: difference Fourier map
*R*[*F*^2^ > 2σ(*F*^2^)] = 0.043	Hydrogen site location: inferred from neighbouring sites
*wR*(*F*^2^) = 0.115	H atoms treated by a mixture of independent and constrained refinement
*S* = 1.04	*w* = 1/[σ^2^(*F*_o_^2^) + (0.0495*P*)^2^ + 0.3428*P*] where *P* = (*F*_o_^2^ + 2*F*_c_^2^)/3
4036 reflections	(Δ/σ)_max_ < 0.001
215 parameters	Δρ_max_ = 0.30 e Å^−^^3^
5 restraints	Δρ_min_ = −0.38 e Å^−^^3^

### Special details

**Table:**

Geometry. All e.s.d.'s (except the e.s.d. in the dihedral angle between two l.s. planes) are estimated using the full covariance matrix. The cell e.s.d.'s are taken into account individually in the estimation of e.s.d.'s in distances, angles and torsion angles; correlations between e.s.d.'s in cell parameters are only used when they are defined by crystal symmetry. An approximate (isotropic) treatment of cell e.s.d.'s is used for estimating e.s.d.'s involving l.s. planes.
Refinement. Refinement of *F*^2^ against ALL reflections. The weighted *R*-factor *wR* and goodness of fit *S* are based on *F*^2^, conventional *R*-factors *R* are based on *F*, with *F* set to zero for negative *F*^2^. The threshold expression of *F*^2^ > σ(*F*^2^) is used only for calculating *R*-factors(gt) *etc*. and is not relevant to the choice of reflections for refinement. *R*-factors based on *F*^2^ are statistically about twice as large as those based on *F*, and *R*- factors based on ALL data will be even larger.

### Fractional atomic coordinates and isotropic or equivalent isotropic displacement parameters (Å^2^)

**Table:**

	*x*	*y*	*z*	*U*_iso_*/*U*_eq_	
P1	0.30253 (4)	0.63760 (6)	0.045212 (19)	0.02129 (13)	
O1W	0.43897 (12)	0.18044 (16)	0.04084 (6)	0.0283 (3)	
H1W	0.5043 (15)	0.175 (3)	0.0196 (8)	0.034*	
H2W	0.4105 (16)	0.072 (2)	0.0412 (8)	0.034*	
N1	0.31522 (13)	0.49375 (19)	−0.01239 (6)	0.0235 (3)	
H1N	0.3457 (17)	0.385 (2)	−0.0046 (8)	0.028*	
O1	0.35590 (11)	0.82247 (16)	0.03322 (5)	0.0273 (3)	
N3	0.15544 (13)	0.6646 (2)	0.06265 (7)	0.0273 (3)	
C1	0.39835 (17)	0.6608 (3)	0.28289 (8)	0.0336 (4)	
H1	0.4026	0.6910	0.3247	0.040*	
C2	0.35064 (16)	0.7858 (3)	0.24143 (8)	0.0314 (4)	
H2	0.3230	0.9034	0.2545	0.038*	
C3	0.34322 (16)	0.7391 (2)	0.18036 (8)	0.0281 (4)	
H3	0.3109	0.8255	0.1518	0.034*	
C4	0.38270 (15)	0.5666 (2)	0.16075 (7)	0.0233 (3)	
N2	0.37225 (13)	0.5161 (2)	0.09875 (6)	0.0243 (3)	
H2N	0.4025 (17)	0.411 (2)	0.0871 (8)	0.029*	
C6	0.26974 (15)	0.5277 (2)	−0.07211 (7)	0.0232 (3)	
C7	0.27217 (15)	0.3843 (2)	−0.11509 (8)	0.0259 (4)	
C8	0.22525 (16)	0.4208 (3)	−0.17281 (8)	0.0312 (4)	
H8	0.2260	0.3251	−0.2022	0.037*	
C9	0.17722 (16)	0.5919 (3)	−0.18920 (8)	0.0327 (4)	
H9	0.1445	0.6125	−0.2289	0.039*	
C10	0.08215 (18)	0.5071 (3)	0.08193 (10)	0.0458 (5)	
H10A	0.0604	0.5220	0.1246	0.069*	
H10B	0.1311	0.3941	0.0771	0.069*	
H10C	0.0055	0.4992	0.0572	0.069*	
C11	0.09013 (19)	0.8401 (3)	0.06091 (9)	0.0403 (5)	
H11A	0.0206	0.8333	0.0316	0.060*	
H11B	0.1480	0.9376	0.0488	0.060*	
H11C	0.0578	0.8679	0.1011	0.060*	
C12	0.22373 (16)	0.7000 (2)	−0.08851 (8)	0.0266 (4)	
H12	0.2236	0.7970	−0.0596	0.032*	
C13	0.17799 (16)	0.7313 (3)	−0.14671 (8)	0.0309 (4)	
H13	0.1469	0.8498	−0.1574	0.037*	
C14	0.32410 (18)	0.1973 (3)	−0.09952 (8)	0.0336 (4)	
H14A	0.3166	0.1163	−0.1349	0.050*	
H14B	0.2773	0.1445	−0.0660	0.050*	
H14C	0.4123	0.2091	−0.0876	0.050*	
C15	0.43306 (15)	0.4396 (2)	0.20245 (8)	0.0257 (4)	
C16	0.47558 (18)	0.2518 (3)	0.18325 (8)	0.0330 (4)	
H16A	0.5079	0.1843	0.2186	0.050*	
H16B	0.5417	0.2642	0.1534	0.050*	
H16C	0.4049	0.1843	0.1652	0.050*	
C17	0.43986 (16)	0.4911 (3)	0.26294 (8)	0.0322 (4)	
H17	0.4742	0.4069	0.2916	0.039*	

### Atomic displacement parameters (Å^2^)

**Table:**

	*U*^11^	*U*^22^	*U*^33^	*U*^12^	*U*^13^	*U*^23^
P1	0.0220 (2)	0.0178 (2)	0.0242 (2)	−0.00113 (16)	0.00295 (16)	−0.00023 (16)
O1W	0.0330 (7)	0.0177 (6)	0.0344 (7)	−0.0005 (5)	0.0081 (5)	−0.0033 (5)
N1	0.0295 (8)	0.0169 (7)	0.0240 (7)	0.0007 (6)	0.0004 (6)	0.0001 (6)
O1	0.0294 (6)	0.0196 (6)	0.0330 (6)	−0.0041 (5)	0.0035 (5)	−0.0014 (5)
N3	0.0232 (7)	0.0259 (8)	0.0331 (8)	0.0014 (6)	0.0046 (6)	0.0030 (6)
C1	0.0321 (10)	0.0433 (11)	0.0252 (9)	−0.0036 (8)	−0.0006 (7)	−0.0073 (8)
C2	0.0260 (9)	0.0334 (10)	0.0347 (10)	−0.0008 (8)	0.0012 (7)	−0.0109 (8)
C3	0.0281 (9)	0.0268 (9)	0.0293 (9)	0.0019 (7)	−0.0020 (7)	−0.0030 (7)
C4	0.0206 (8)	0.0248 (9)	0.0244 (8)	−0.0026 (7)	0.0011 (6)	−0.0020 (7)
N2	0.0283 (7)	0.0203 (7)	0.0244 (7)	0.0022 (6)	0.0012 (6)	−0.0042 (6)
C6	0.0209 (8)	0.0245 (9)	0.0242 (8)	−0.0050 (6)	0.0022 (6)	0.0004 (7)
C7	0.0227 (8)	0.0264 (9)	0.0287 (9)	−0.0049 (7)	0.0037 (7)	−0.0035 (7)
C8	0.0286 (9)	0.0384 (11)	0.0267 (9)	−0.0056 (8)	0.0025 (7)	−0.0057 (8)
C9	0.0270 (9)	0.0453 (12)	0.0258 (9)	−0.0048 (8)	−0.0017 (7)	0.0039 (8)
C10	0.0289 (10)	0.0475 (13)	0.0613 (14)	−0.0055 (9)	0.0109 (9)	0.0170 (11)
C11	0.0372 (11)	0.0417 (12)	0.0422 (11)	0.0157 (9)	0.0084 (9)	0.0062 (9)
C12	0.0268 (9)	0.0239 (9)	0.0293 (9)	−0.0037 (7)	−0.0001 (7)	−0.0001 (7)
C13	0.0278 (9)	0.0299 (10)	0.0350 (10)	−0.0041 (7)	−0.0007 (7)	0.0078 (8)
C14	0.0374 (10)	0.0297 (10)	0.0338 (10)	0.0008 (8)	−0.0007 (8)	−0.0085 (8)
C15	0.0202 (8)	0.0267 (9)	0.0302 (9)	−0.0030 (7)	0.0002 (7)	−0.0004 (7)
C16	0.0391 (10)	0.0288 (10)	0.0310 (9)	0.0033 (8)	−0.0018 (8)	0.0042 (7)
C17	0.0295 (9)	0.0391 (11)	0.0278 (9)	−0.0023 (8)	−0.0035 (7)	0.0006 (8)

### Geometric parameters (Å, º)

**Table:**

P1—O1	1.4833 (12)	C7—C14	1.504 (2)
P1—N3	1.6392 (14)	C8—C9	1.389 (3)
P1—N2	1.6439 (15)	C8—H8	0.9500
P1—N1	1.6530 (14)	C9—C13	1.380 (3)
O1W—H1W	0.850 (14)	C9—H9	0.9500
O1W—H2W	0.841 (14)	C10—H10A	0.9800
N1—C6	1.420 (2)	C10—H10B	0.9800
N1—H1N	0.871 (14)	C10—H10C	0.9800
N3—C11	1.453 (2)	C11—H11A	0.9800
N3—C10	1.454 (2)	C11—H11B	0.9800
C1—C2	1.381 (3)	C11—H11C	0.9800
C1—C17	1.383 (3)	C12—C13	1.387 (2)
C1—H1	0.9500	C12—H12	0.9500
C2—C3	1.392 (2)	C13—H13	0.9500
C2—H2	0.9500	C14—H14A	0.9800
C3—C4	1.392 (2)	C14—H14B	0.9800
C3—H3	0.9500	C14—H14C	0.9800
C4—C15	1.404 (2)	C15—C17	1.388 (2)
C4—N2	1.420 (2)	C15—C16	1.500 (2)
N2—H2N	0.869 (14)	C16—H16A	0.9800
C6—C12	1.389 (2)	C16—H16B	0.9800
C6—C7	1.409 (2)	C16—H16C	0.9800
C7—C8	1.388 (2)	C17—H17	0.9500
			
O1—P1—N3	107.97 (7)	C13—C9—H9	120.7
O1—P1—N2	116.20 (7)	C8—C9—H9	120.7
N3—P1—N2	108.73 (7)	N3—C10—H10A	109.5
O1—P1—N1	113.35 (7)	N3—C10—H10B	109.5
N3—P1—N1	110.37 (7)	H10A—C10—H10B	109.5
N2—P1—N1	99.98 (7)	N3—C10—H10C	109.5
H1W—O1W—H2W	105.3 (15)	H10A—C10—H10C	109.5
C6—N1—P1	125.07 (12)	H10B—C10—H10C	109.5
C6—N1—H1N	117.5 (12)	N3—C11—H11A	109.5
P1—N1—H1N	117.1 (12)	N3—C11—H11B	109.5
C11—N3—C10	115.75 (15)	H11A—C11—H11B	109.5
C11—N3—P1	124.21 (12)	N3—C11—H11C	109.5
C10—N3—P1	120.03 (12)	H11A—C11—H11C	109.5
C2—C1—C17	119.38 (17)	H11B—C11—H11C	109.5
C2—C1—H1	120.3	C13—C12—C6	120.50 (17)
C17—C1—H1	120.3	C13—C12—H12	119.8
C1—C2—C3	119.80 (17)	C6—C12—H12	119.8
C1—C2—H2	120.1	C9—C13—C12	120.55 (18)
C3—C2—H2	120.1	C9—C13—H13	119.7
C4—C3—C2	120.53 (17)	C12—C13—H13	119.7
C4—C3—H3	119.7	C7—C14—H14A	109.5
C2—C3—H3	119.7	C7—C14—H14B	109.5
C3—C4—C15	120.06 (15)	H14A—C14—H14B	109.5
C3—C4—N2	120.83 (15)	C7—C14—H14C	109.5
C15—C4—N2	119.11 (15)	H14A—C14—H14C	109.5
C4—N2—P1	125.66 (12)	H14B—C14—H14C	109.5
C4—N2—H2N	119.2 (12)	C17—C15—C4	117.90 (16)
P1—N2—H2N	115.1 (12)	C17—C15—C16	120.38 (16)
C12—C6—C7	119.93 (15)	C4—C15—C16	121.70 (15)
C12—C6—N1	120.84 (15)	C15—C16—H16A	109.5
C7—C6—N1	119.23 (15)	C15—C16—H16B	109.5
C8—C7—C6	117.91 (17)	H16A—C16—H16B	109.5
C8—C7—C14	120.57 (16)	C15—C16—H16C	109.5
C6—C7—C14	121.52 (15)	H16A—C16—H16C	109.5
C7—C8—C9	122.43 (17)	H16B—C16—H16C	109.5
C7—C8—H8	118.8	C1—C17—C15	122.30 (18)
C9—C8—H8	118.8	C1—C17—H17	118.8
C13—C9—C8	118.67 (17)	C15—C17—H17	118.8
			
O1—P1—N1—C6	−55.73 (15)	P1—N1—C6—C7	−171.82 (12)
N3—P1—N1—C6	65.52 (15)	C12—C6—C7—C8	−1.3 (2)
N2—P1—N1—C6	179.92 (13)	N1—C6—C7—C8	178.99 (14)
O1—P1—N3—C11	4.80 (17)	C12—C6—C7—C14	178.50 (16)
N2—P1—N3—C11	131.68 (15)	N1—C6—C7—C14	−1.2 (2)
N1—P1—N3—C11	−119.60 (15)	C6—C7—C8—C9	0.3 (2)
O1—P1—N3—C10	−174.57 (15)	C14—C7—C8—C9	−179.55 (16)
N2—P1—N3—C10	−47.69 (17)	C7—C8—C9—C13	1.0 (3)
N1—P1—N3—C10	61.04 (16)	C7—C6—C12—C13	1.1 (2)
C17—C1—C2—C3	0.9 (3)	N1—C6—C12—C13	−179.17 (15)
C1—C2—C3—C4	0.5 (3)	C8—C9—C13—C12	−1.2 (3)
C2—C3—C4—C15	−1.4 (2)	C6—C12—C13—C9	0.1 (3)
C2—C3—C4—N2	178.28 (15)	C3—C4—C15—C17	1.0 (2)
C3—C4—N2—P1	−6.6 (2)	N2—C4—C15—C17	−178.72 (14)
C15—C4—N2—P1	173.12 (12)	C3—C4—C15—C16	179.43 (16)
O1—P1—N2—C4	61.98 (15)	N2—C4—C15—C16	−0.2 (2)
N3—P1—N2—C4	−60.03 (15)	C2—C1—C17—C15	−1.3 (3)
N1—P1—N2—C4	−175.68 (13)	C4—C15—C17—C1	0.4 (3)
P1—N1—C6—C12	8.5 (2)	C16—C15—C17—C1	−178.08 (17)

### Hydrogen-bond geometry (Å, º)

**Table:**

*D*—H···*A*	*D*—H	H···*A*	*D*···*A*	*D*—H···*A*
O1*W*—H2*W*···O1^i^	0.84 (1)	1.91 (2)	2.7491 (17)	173 (2)
O1*W*—H1*W*···O1^ii^	0.85 (1)	1.91 (1)	2.7607 (17)	175 (2)
N1—H1*N*···O1*W*	0.87 (1)	2.04 (2)	2.8724 (19)	159 (2)
N2—H2*N*···O1*W*	0.87 (1)	2.00 (2)	2.8473 (18)	164 (2)

Symmetry codes: (i) *x*, *y*−1, *z*; (ii) −*x*+1, −*y*+1, −*z*.
